# Supplementary material for: Effects of Molecular Crowding and Betaine on HSPB5 Interactions, with Target Proteins Differing in the Quaternary Structure and Aggregation Mechanism
Source: Int J Mol Sci. 2022 Dec 6;23(23):15392. doi: 10.3390/ijms232315392 (PMC9737104; doi:10.3390/ijms232315392)
Supplement: Supplementary file 1 [file ijms-23-15392-s001.zip › ijms-2072776-supplementary.pdf]

Supplementary Materials

Effect of Molecular Crowding and an Osmolyte on HspB5 Interaction with Target Proteins Differing in the Quaternary Structure and Aggregation Mechanism

Vera. A. Borzova, Svetlana G. Roman, Anastasiya V. Pivovarova and Natalia A. Chebotareva*

Bach Institute of Biochemistry, Federal Research Centre "Fundamentals of Biotechnology" of the Russian Academy of Sciences, Leninsky pr. 33, 119071 Moscow, Russia

* Correspondence: n.a.chebotareva@gmail.com

S1. The kinetics of thermal aggregation of GDH and DTT-induced aggregation of Lyz

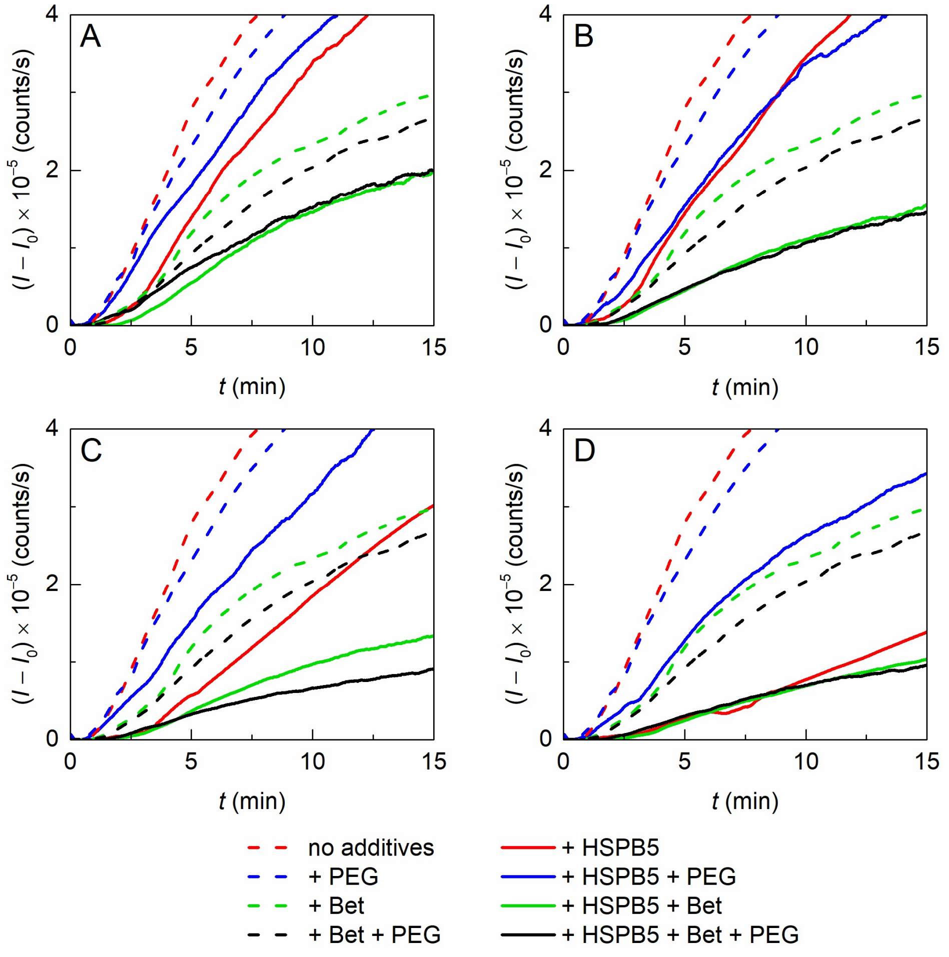

Figure S1. The dependences of the light scattering intensity on time for the heat-induced aggregation of GDH (0.2 mg/mL) at 50 °C in the absence and in the presence of the osmolyte (1 M Bet), the crowding agent (25 mg/mL PEG) and their mixture at the following concentrations of HSPB5: (**A**) 0.01 mg/mL, (**B**) 0.025 mg/mL, (**C**) 0.05 mg/mL, (**D**) 0.1 mg/mL.

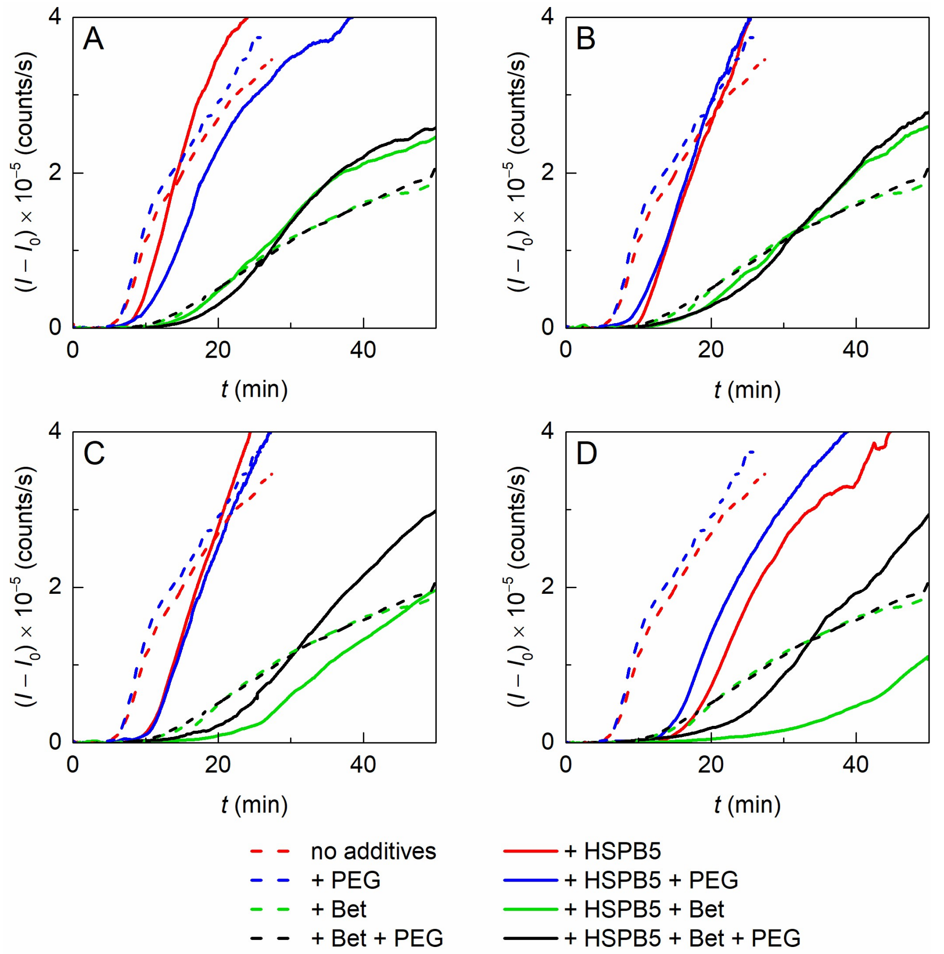

Figure S2. The dependences of the light scattering intensity on time for the DTT-induced aggregation of Lyz (0.15 mg/mL) at 37 °C in the absence and in the presence of the osmolyte (1 M Bet), the crowding agent (25 mg/mL PEG20000) and their mixture at the following concentrations of HSPB5: (**A**) 0.01 mg/mL, (**B**) 0.025 mg/mL, (**C**) 0.05 mg/mL, (**D**) 0.1 mg/mL.

S2. The sedimentation behavior of GDH and Lyz in the presence of osmolyte, crowding agent and mixed crowding

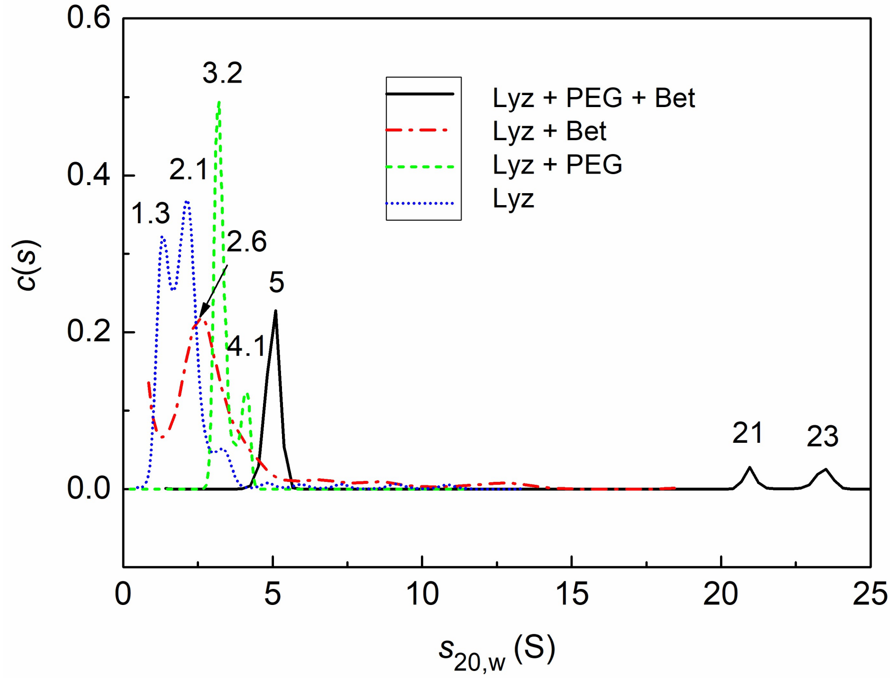

Figure S3. The sedimentation behavior of Lyz (0.15 mg/mL) in the presence of the osmolyte, the crowding agent and their mixture. The differential sedimentation coefficient distributions, c(s), for Lyz (blue dotted curve) with the addition of 25 mg/mL PEG (green dashed curve), 1 M Bet (red dash-dotted curve), 25 mg/mL PEG + 1 M Bet (black solid curve). All *c*(*s*) distributions were obtained at 37 °C and corrected to standard conditions. The rotor speed was 60,000 rpm in the case of Lyz and (Lyz + PEG), and 48,000 rpm for (Lyz + Bet) and (Lyz+PEG+Bet) mixtures.

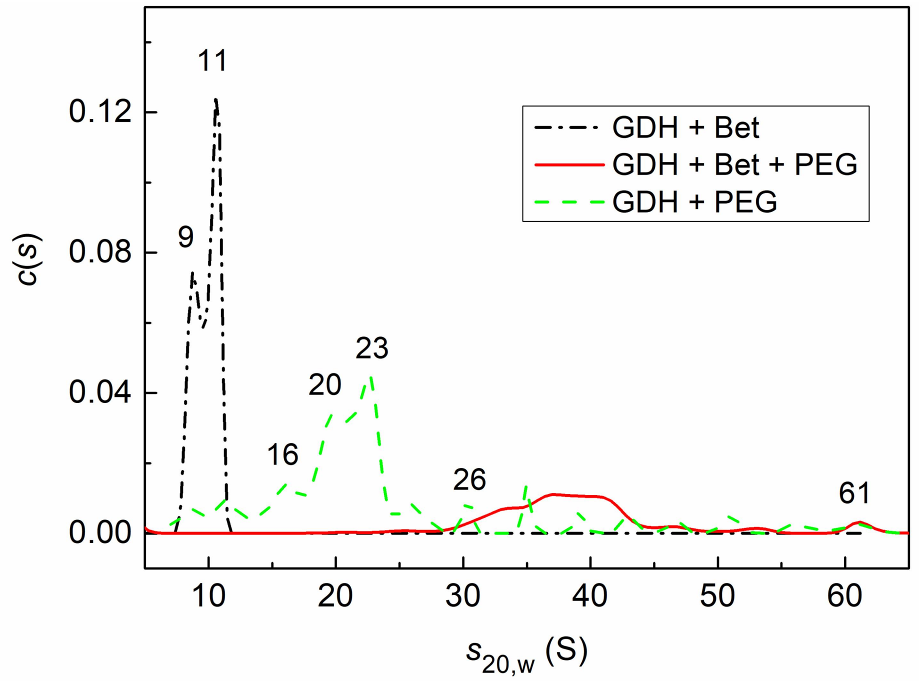

Figure S4. Effect of the osmolyte, the crowding agent and their mixture on the oligomeric state of GDH (0.2 mg/mL) at 25 °C. Samples were pre-heated 10 min at 50 °C prior to the experiment. The differential sedimentation coefficient distributions, c(s), distributions for GDH in the presence of 1 M Bet (black dash-dotted curve), 25 mg/mL PEG (green dashed curve), and their mixture (red solid curve) were obtained at 25 °C and transformed to standard conditions. The rotor speed was 48,000 rpm.

S3. The size of GDH aggregates in the absence and in the presence of HSPB5

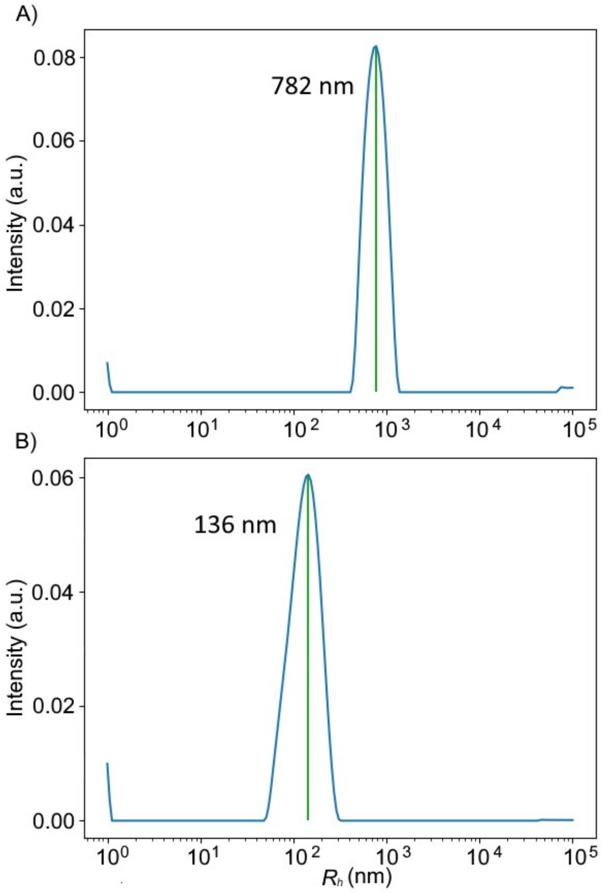

Figure S5. The intensity distributions of R_h for GDH (0.2 mg/mL) after 10 min of aggregation at 50 °C in the absence (**A**) and in the presence (**B**) of 0.05 mg/mL HSPB5. The calculations were carried out at dls.rogach.org using values of dynamic viscosity η = 0.5832 and refractive index n = 1.33098 for 0.1 M Na-phosphate buffer, pH 7.6, 10 mM NaCl at 50 °C.

S4. Density and dynamic viscosity of PEG and Bet solutions

Table S1. The values of density (ϱ) and dynamic viscosity (η) of PEG and Bet in 0.1 M Na-phosphate buffer (pH 7.6, 10 mM NaCl) at 25 and 37 °C.

Additive	ϱ (g/cm3)	η (mPa·s)
**25 °C**		
None	1.0123 ± 0.0005	0.945 ± 0.001
1 M Bet	1.0204 ± 0.0005	1.142 ± 0.001
25 mg/mL PEG	1.0168 ± 0.0005	2.099 ± 0.001
1 M Bet + 25 mg/mL PEG	1.0248 ± 0.0005	2.385 ± 0.001
**37 °C**		
None	1.0065 ± 0.0005	0.738 ± 0.001
1 M Bet	1.0156 ± 0.0005	0.879 ± 0.001
25 mg/mL PEG	1.0104 ± 0.0005	1.560 ± 0.001
1 M Bet + 25 mg/mL PEG	1.0243 ± 0.0005	1.768 ± 0.001
